# Caesarean section in four South East Asian countries: reasons for, rates, associated care practices and health outcomes

**DOI:** 10.1186/1471-2393-9-17

**Published:** 2009-05-09

**Authors:** Mario R Festin, Malinee Laopaiboon, Porjai Pattanittum, Melissa R Ewens, David J Henderson-Smart, Caroline A Crowther, The SEA-ORCHID Study Group

**Affiliations:** 1 Department of Obstetrics and Gynecology, College of Medicine, University of the Philippines Manila, Manila 1000, Philippines; 2 Department of Biostatistics and Demography, Khon Kaen University, Khon Kaen 40002, Thailand; 3 Discipline of Obstetrics and Gynaecology, University of Adelaide, Adelaide, SA 5006, Australia; 4 NSW Centre for Perinatal Health Services Research, Queen Elizabeth ll Research Institute, University of Sydney, Sydney, NSW 2006, Australia

## Abstract

**Background:**

Caesarean section is a commonly performed operation on women that is globally increasing in prevalence each year. There is a large variation in the rates of caesarean, both in high and low income countries, as well as between different institutions within these countries. This audit aimed to report rates and reasons for caesarean and associated clinical care practices amongst nine hospitals in the four South East Asian countries participating in the South East Asia-Optimising Reproductive and Child Health in Developing countries (SEA-ORCHID) project.

**Methods:**

Data on caesarean rates, care practices and health outcomes were collected from the medical records of the 9550 women and their 9665 infants admitted to the nine participating hospitals across South East Asia between January and December 2005.

**Results:**

Overall 27% of women had a caesarean section, with rates varying from 19% to 35% between countries and 12% to 39% between hospitals within countries. The most common indications for caesarean were previous caesarean (7.0%), cephalopelvic disproportion (6.3%), malpresentation (4.7%) and fetal distress (3.3%). Neonatal resuscitation rates ranged from 7% to 60% between countries. Prophylactic antibiotics were almost universally given but variations in timing occurred between countries and between hospitals within countries.

**Conclusion:**

Rates and reasons for caesarean section and associated clinical care practices and health outcomes varied widely between the four South East Asian countries.

## Background

Caesarean section is a commonly performed operation on women that is globally increasing in prevalence each year [1-5]. There is a large variation in the rates of caesarean, both in high and low income countries, as well as between different institutions within these countries [3,4].

In the past, recommended caesarean rates have been calculated using various methods and concepts, the most common of which is based on the number of births in a hospital. The most widely recommended upper limit rate of caesarean section was 15 percent as advocated by the World Health Organization (WHO) [6]. This was based on caesarean rates of countries with the lowest maternal and neonatal mortality rate at the time of the recommendation, and took into account both developed and developing countries [4,6]. Since then the World Health Organization has published a revision in 1994, stating that acceptable caesarean section rates should range between 5 and 15 percent [7].

Caesarean section in developing countries is associated with significant increases in maternal morbidity [4,8] particularly following elective caesarean section [9] and caesarean section without medical indications [10]. Increases in infant morbidity and mortality are associated with caesareans in developing countries [3,4,10]. However, in low income countries, very low caesarean rates (less than 1%) have been associated with higher maternal and infant mortality linked to the inability to perform a caesarean section when needed [4,11].

Interventions aimed at reducing maternal and perinatal morbidity and mortality associated with caesarean have included auditing of the rates, indications for and associated health outcomes [12,13], while interventions to reduce high caesarean rates and inappropriate caesarean practices have involved the use of best evidence such as in the WHO Reproductive Health Library [14] and mandatory second opinion for non-emergency caesarean section [15].

The SEA-ORCHID (South East Asia – Optimising Reproductive and Child Health in Developing countries) project [16] across four South East Asian countries found the average rate of caesarean section to be 27% [17]. We therefore conducted this review of caesarean section practice in hospitals within the countries participating in SEA-ORCHID to assess information on the rates, reasons for and care practices associated with caesarean. We also looked at the pregnancy background of women and health outcomes for women who had a caesarean and their babies.

## Methods

### Setting

Nine hospitals participating in the SEA-ORCHID project representing different types of hospitals across four countries in South East Asia (Indonesia, Malaysia, The Philippines and Thailand) were audited, with support from three sites in Australia [17]. The SEA-ORCHID project settings and methods have been published elsewhere [16].

Seven of the nine hospitals were tertiary (university and regional) referral institutions with regional referrals of women with a high risk pregnancy and two were provincial or district institutions. The hospital delivery care models included a multidisciplinary approach with midwives (including nurses with midwifery qualifications) or obstetric specialists. Caesarean section facilities and obstetric specialists were available, and doctors and/or midwives (including nurses with midwifery qualifications) conducted normal vaginal births in all hospitals.

Approval for the project was given by the local ethics committee of each hospital and by the ethics committee of the University of Sydney, the administering institution in Australia

### Procedure

As part of the SEA-ORCHID project baseline data collection, between January and December 2005, we previously reviewed the medical records of 9550 women and their 9665 infants (including 111 twins and two sets of triplets) admitted to the labour wards at the nine participating hospitals. Data were collected on a consecutive basis at five of the participating hospitals until a total of at least 1000 women's medical records had been reviewed. Cases were sampled using a variety of ratios at the four largest hospitals. This method was used to ensure data were collected for a minimum of three months from each hospital and over similar time periods.

For the current audit, medical records were reviewed by trained staff using pre-established and piloted data extraction forms. Information about women who gave birth by caesarean section and their babies was collected.

Main indications for caesarean section were collected by trained staff who selected a single main reason for caesarean section from a predetermined list (Table 1)

**Table 1:** Rates and main indications for caesarean section (as percentage of overall births and percentages of rates consecutively)

	**Indonesia**			**Malaysia**			**The Philippines**			**Thailand**			
	
	**Overall**	Tertiary	District	**Overall**	Tertiary 1	Tertiary 2	**Overall**	Tertiary 1	Tertiary 2	**Overall**	Regional	University	Provincial
	
	**n = 2086**	n = 1019	n = 1067	**n = 2379**	n = 1249	n = 1130	**n = 2085**	n = 1026	n = 1059	**n = 3000**	n = 1000	n = 1000	n = 1000
**Rate of caesarean section**	**29.6**	28.7	30.6	**19.1**	21.1	16.8	**22.7**	12.3	32.9	**34.8**	33.3	33.2	38.0

**Indication for caesarean section**													

**Malpresentations**	**5.5**	3.8	7.1	**5.0**	5.4	4.6	**3.9**	2.5	5.3	**4.5**	5.5	3.8	4.3

**Previous caesarean section**	**4.5**	3.0	5.8	**3.3**	3.5	3.1	**10.1**	6.6	13.4	**9.7**	8.8	9.3	11.0

**Cephalopelvic disproportion**	**3.8**	2.0	5.5	**4.8**	5.4	4.2	**3.0**	1.3	4.6	**11.4**	9.7	9.3	15.2

**Fetal distress**	**3.3**	4.0	2.6	**3.3**	3.8	2.7	**2.0**	0.3	3.7	**4.2**	5.2	3.8	3.7

**Failure to progress**	**3.4**	4.3	2.4	**0.0**	0.0	0.0	**0.0**	0.0	0.0	**0.7**	0.2	1.4	0.6

**Antepartum haemorrhage**	**2.5**	2.5	2.5	**1.2**	1.2	1.2	**1.7**	1.0	2.5	**0.6**	0.8	0.6	0.5

**Pre-eclampsia/eclampsia**	**2.2**	3.2	1.1	**0.0**	0.0	0.0	**0.0**	0.0	0.0	**0.7**	1.1	0.4	0.7

**Maternal request**	**2.1**	3.7	0.6	**0.0**	0.0	0.0	**0.0**	0.0	0.0	**0.0**	0.0	0.0	0.0

**Premature rupture of membranes**	**1.6**	1.0	2.2	**0.0**	0.0	0.0	**0.0**	0.0	0.0	**0.3**	0.2	0.3	0.3

**Other Maternal conditions**	**0.4**	0.4	0.5	**0.7**	1.0	0.4	**1.0**	0.0	1.9	**1.4**	0.6	2.9	0.8

**Multiple pregnancy**	**0.0**	0.0	0.0	**0.1**	0.1	0.2	**0.2**	0.1	0.3	**0.7**	0.6	0.6	0.9

**Other**	**0.4**	0.7	0.2	**0.5**	0.6	0.4	**0.9**	0.5	1.2	**0.5**	0.6	0.8	0.0

Figures are percentage rounded to one decimal point

Other information collected from the medical record regarding maternal and perinatal care practice around caesarean section included prophylactic antibiotic use and estimated blood loss for women having a caesarean section (Table 2) and use of antibiotics during postnatal care for women (Table 3).

**Table 2:** Use of prophylactic antibiotics and blood loss for women having a caesarean section (as percentage of caesarean deliveries)

	**Indonesia**			**Malaysia**			**The Philippines**			**Thailand**			
	
	**Overall**	Tertiary	District	**Overall**	Tertiary 1	Tertiary 2	**Overall**	Tertiary 1	Tertiary 2	**Overall**	Regional	University	Provincial
	
	**n = 618**	n = 292	n = 326	**n = 453**	n = 264	n = 189	**n = 474**	n = 126	n = 348	**n = 1045**	n = 333	n = 332	n = 380
**Antibiotics given**	**100**	100	100	**99**	100	98	**93**	100	91	**100**	100	99	100

**If yes, when given**													
pre-operatively	**0**	0	0	**60**	100	4	**58**	99	41	**9**	21	2	6
after cord clamped	**0**	0	0	**1**	0	1	**12**	1	16	**88**	78	99	87
post-operatively	**100**	100	100	**39**	0	95	**31**	**0**	**43**	**3**	1	0	7

**If given, which antibiotics**													
cephalosporin	**77**	69	84	**59**	100	1	**73**	82	69	**84**	76	94	83
ampicillin	**7**	15	0	**37**	0	90	**4**	5	3	**13**	22	5	11
other	**16**	16	16	**4**	0	9	**23**	14	27	**3**	2	1	6

**If given, what dosage**													
single	**0**	0	0	**59**	100	0	**76**	93	70	**53**	82	84	1
multiple	**100**	100	100	**41**	0	100	**24**	7	30	**47**	18	16	99

**Blood loss at caesarean section**													
< = 500 mls	**96**	95	97	**74**	70	80	**21**	35	17	**67**	72	43	83
501 – 1000 mls	**4**	5	3	**19**	22	14	**75**	62	80	**32**	26	56	17
≥ 1000 mls	**0**	0	0	**7**	8	6	**4**	3	4	**1**	2	1	0

**Postpartum haemorrhage > 500 ml**	**4**	5	3	**26**	30	20	**79**	65	83	**33**	28	57	17

**Postpartum transfusion**	**5**	4	6	**9**	8	10	**4**	3	4	**1**	1	1	0

Figures are percentage rounded to the nearest whole number

**Table 3:** Use of antibiotics during postnatal care for women (as percentage of caesarean deliveries)

	**Indonesia**			**Malaysia**			**The Philippines**			**Thailand**			
	
	**Overall**	Tertiary	District	**Overall**	Tertiary 1	Tertiary 2	**Overall**	Tertiary 1	Tertiary 2	Overall	Regional	University	Provincial
	
	**n = 619**	n = 292	n = 327	**n = 454**	n = 264	n = 190	**n = 474**	n = 126	n = 348	**n = 1045**	n = 333	n = 332	n = 380
**Antibiotics postpartum**	**100**	100	100	**41**	3	94	**54**	98	38	**48**	24	15	97

**If yes, antibiotics given for**													
Prophylaxis	**100**	100	100	**90**	0	93	**36**	0	69	**90**	71	67	97
Wound infection	**0**	0	0	**0**	0	0	**52**	98	8	**0**	1	4	0
Preterm prelabour rupture of membranes	**0**	0	0	**6**	43	4	**0**	0	0	**4**	11	4	2
Urinary tract infection	**0**	0	0	**2**	43	0	**0**	0	0	**1**	1	4	0
Endometritis	**0**	0	0	**2**	14	1	**0**	0	0	**2**	4	8	1
Upper respiratory tract infection	**0**	0	0	**1**	0	1	**0**	1	0	**1**	6	4	0
Other/Unknown	**0**	0	0	**1**	0	1	**12**	1	22	**2**	6	8	0

Figures are percentage rounded to the nearest whole number

Health outcomes for infants born by caesarean section were collected and included gestational age at birth, birth weight, need for resuscitation, low Apgar scores at 1 and 5 minutes, stillbirth, babies born alive who later died and total death rates (Table 4).

**Table 4:** Health outcomes for infants who were born by caesarean section (as percentage of caesarean born babies)

	**Indonesia**			**Malaysia**			**The Philippines**			**Thailand**			
	
	**Overall**	Tertiary	District	**Overall**	Tertiary 1	Tertiary 2	**Overall**	Tertiary 1	Tertiary 2	**Overall**	Regional	University	Provincial
	
	**n = 628**	n = 294	n = 334	**n = 465**	n = 272	n = 193	**n = 479**	n = 126	n = 353	**n = 1074**	n = 342	n = 342	n = 390
**Stillbirth **^#^	**1.0**	2.0	0.0	**0.9**	0.4	1.6	**0.2**	0.8	0.0	**0.4**	0.0	0.6	0.5

**Babies born alive who died **#	**0.3**	0.0	0.6	**0.4**	0.4	0.5	**0.4**	0.8	0.3	**1.1**	0.9	1.5	1.0

**Total deaths **^#^	**1.3**	2.0	0.6	**1.3**	0.7	2.1	**0.6**	1.6	0.3	**1.5**	0.9	2.0	1.5

**Gestational age at birth **(weeks) *	**38.8 ** **(2.2)**	38.3 (2.7)	39.2 (1.4)	**37.9 ** **(2.0)**	37.8 (2.2)	38.3 (1.7)	**37.8 ** **(2.1)**	38.1 (1.5)	37.7 (2.2)	**38.3 ** **(2.2)**	37.9 (2.3)	38.1 (2.3)	38.8 (2.0)

**Gestational age at birth < 37 weeks**	**10**	18	3	**13**	15	11	**10**	3	12	**12**	16	13	9

**Birth weight **(kg) *	**2.97 ** **(0.65)**	2.87 (0.74)	3.01 (0.56)	**3.02 ** **(0.70)**	3.0 (0.71)	3.05 (0.69)	**2.8 ** **(0.60)**	2.89 (0.48)	2.76 (0.63)	**3.06 ** **(0.60)**	3.0 (0.58)	3.1 (0.63)	3.13 (0.57)
very low birth weight (< 1500 g)	**3**	5	1	**3**	3	3	**3**	1	4	**2**	2	3	1
low birth weight (1500–2499 g)	**16**	20	13	**15**	15	15	**21**	15	24	**10**	16	8	7
normal (2500–4499 g)	**77**	70	83	**74**	74	75	**75**	85	71	**84**	79	86	87
macrosomia (≥ 4000 g)	**5**	5	4	**8**	7	8	**1**	0	1	**4**	4	3	5

**Resuscitation**	**43**	40	46	**7**	5	10	**16**	19	15	**60**	21	57	97

**Apgar score < 7 at 1 min**^§^	**35**	42	28	**8**	7	9	**9**	8	10	**6**	7	7	4

**Apgar score < 7 at 5 min**^§^	**9**	14	5	**2**	2	3	**3**	2	4	**2**	2	2	2

Figures are percentage rounded to the nearest whole number, or ^#^percentagerounded to one decimal point or *mean (standard deviation)

^§^Calculated for live births only

Trained fieldworkers used a secure web-based database to manually enter the data. The online form allowed validation checks to be performed to detect discrepancies and missing data and thus ensured transcription errors were minimized.

### Data analysis

Data analysis was performed using STATA software Version 8.0 [18]. Descriptive analysis was performed between hospitals within countries as well as across countries. For categorical data, frequencies were used to describe maternal characteristics, maternal and perinatal care practices and birth outcomes. For continuous data, means and standard deviations (SDs) were used.

### Ethics Approval

The SEA-ORCHID project was approved by the local ethics committees of each hospital and by the ethics committee of the University of Sydney, the administering institution in Australia.

## Results

Of the 9550 women, 2592 (27%) women and their 2645 (27%) babies were born by caesarean. Actual rates varied from 12% to 39% between hospitals and from 19% to 35% between countries (Table 1).

### Rates and indications for caesarean section (Table 1)

The most common indications for caesarean were malpresentation, previous caesarean section, cephalopelvic disproportion, and fetal distress. In Indonesia and Malaysia, the most common indication was malpresentation with rates of 5.5% and 5.0% respectively. In The Philippines, caesarean in a previous pregnancy was the most common indication for a caesarean for mothers who gave birth again (10.1%), while cephalopelvic disproportion was the most frequent indication in Thailand (11.4%).

Common pregnancy complications such as preeclampsia and antepartum haemorrhage were not often given as indications for caesarean. Although maternal request for a caesarean was relatively frequent in one of the tertiary hospitals in Indonesia (3.7%), this was not an indication in Malaysia, Thailand and The Philippines.

### Prophylactic antibiotic use for mothers who gave birth by caesarean section (Table 2)

Prophylactic antibiotics were almost universally given across all four countries in South East Asia, with only one tertiary hospital in The Philippines reporting a slightly lower rate of 91%. There was variation in the timing of prophylactic antibiotics, both between countries and between hospitals within countries. In Indonesia, prophylactic antibiotics were universally given post-operatively. In one Malaysian hospital they were always given pre-operatively, while in the other they were given post-operatively 95% of the time. In one hospital in The Philippines, mothers were given prophylactic antibiotics pre-operatively almost universally, while in the other hospital 41% of mothers received antibiotics pre-operatively and 43% post-operatively, with the remainder given intra-operatively after umbilical cord clamping. In Thailand almost 90% of women were given prophylactic antibiotics intra-operatively after umbilical cord clamping, with the next most common time of administration being pre-operatively.

Cephalosporin was the most common class of prophylactic antibiotics used across all hospitals with a rate of 73%. Ampicillin was the next most commonly used antibiotic in Malaysia and Thailand, while 'other' antibiotics were the next most common in Indonesia and The Philippines. The frequency of dose for prophylactic antibiotics varied both between countries and between hospitals in countries. Mothers in Indonesia received multiple doses of prophylactic antibiotics while mothers in Malaysia received either a single dose or multiple doses depending on their births or the hospital. In The Philippines and Thailand rates ranged from 1% to 93% for single doses of prophylactic antibiotics and from 7% to 99% for multiple doses.

### Caesarean section and blood loss (Table 2)

In Indonesia, Malaysia, and Thailand, the majority of women were reported to have a less than 500 ml estimated blood loss, while in the Philippines 79% were estimated to have a greater than 500 ml blood loss. The reported postpartum haemorrhage rate > 500 ml for Indonesia was only 4%. Malaysia reported the highest rate for postpartum maternal transfusion (9%).

### Postnatal care after caesarean section (Table 3)

Mothers were often given prophylactic antibiotics postnatally with rates varying between countries and between hospitals within countries. All mothers in Indonesia were given prophylactic antibiotics postnatally. Rates varied widely between hospitals in Malaysia (3% and 94%), The Philippines (38% and 98%) and Thailand (15% to 97%). The main reason for giving antibiotics postnatally to women was prophylaxis and this was commonly practiced in Indonesia (100%), Thailand (90%) and Malaysia (90%), although it was less common in The Philippines (36%) where wound infection was the main reason reported (52%) for postnatal antibiotic administration.

### Birth and infant health outcomes (Table 4)

The mean gestational age at birth of babies born by caesarean across the hospitals was similar (range 37.7 (SD 2.2) to 39.2 (SD 1.4) weeks). The preterm birth rate (< 37 weeks gestation) varied widely from 3% to 18% between hospitals although similar from 10% to 13% between countries. Overall, 16% of the babies born by caesarean were of low birth weight (< 2500 g), with rates ranging from 8% to 28% between hospitals. The mean birth weight of babies ranged from 2.76 kg (SD 0.63) to 3.13 kg (SD 0.57) between hospitals and 2.8 kg (SD 0.60) to 3.06 kg (SD 0.60) between countries.

There were wide variations in the use of neonatal resuscitation at caesarean. Babies born by caesarean in Thailand received resuscitation in 60% of cases, however actual rates varied widely between hospitals, ranging from 21% in the regional hospital to 97% in the provincial hospital. In Malaysia, only 7% of babies born by caesarean received resuscitation. Rates of babies with Apgar scores < 7 at 5 minutes were higher in the two Indonesian hospitals compared with hospitals in the other three South East Asian countries (5% and 12% compared with range 1% to 4%).

The rates for caesarean section where the baby was stillborn ranged between 0% to 1% between countries and 0% to 2% between hospitals within countries. The rates for babies born alive by caesarean who then died were reported as 0% overall for Indonesia, Malaysia and The Philippines, with these three countries recording a rate of 1% in one hospital each, while Thailand recorded a rate of 1% in all hospitals as well as overall.

## Discussion

### Caesarean section rates in South East Asian countries

Actual caesarean rates in developing countries, including South East Asia, are largely unknown because of a lack of reliable data. Our results showed the overall caesarean rates, for all hospitals and all countries in the audit, to be 27%, higher than the WHO recommended rates of between 5 and 15 percent [7]. This may be attributable to the fact that most of the hospitals audited were referral centres, meaning a higher proportion of women with complications from other lower category hospitals would have been sent to these hospitals. Some of the variation in caesarean section rates between hospitals may be related to differing maternal characteristics. Regardless, the caesarean rates in the South East Asian countries and hospitals audited are higher than the nationally representative data available [19].

### Main indications for caesarean section

Women in the four South East Asian countries audited were more likely to have a caesarean if they or their infant experienced malpresentation, previous caesarean section, cephalopelvic disproportion or fetal distress. The National Collaborating Centre for Women's and Children's Health (NCCWCH) with The Royal College of Obstetricians and Gynaecologists (RCOG) [2] guidelines list malpresentation, cephalopelvic disproportion and fetal distress as main indicators for caesarean section, consistent with the indications in our population. Trained staff coded the main reasons for caesarean section, therefore we consider misclassification unlikely to account for variation between institutions.

Previous caesarean section as an indication for caesarean section is not a recommendation of the NCCWCH/RCOG UK guidelines [2]. The high incidence of this as an indicator for caesarean section in the four South East Asian countries could be due to women or providers choosing this option after a previous complicated birth; a scenario more common in developing countries [20]. In addition, women may not be fully informed and educated about vaginal birth and associated pain and management [21]. High caesarean rates may be attributed to limited knowledge and training of health professionals in developing countries causing limited implementation of recommendations such as vaginal birth after caesarean (VBAC). It is known that maternal morbidity particularly increases following elective caesarean section [9] and caesarean section without medical indications [10] in developing countries. It is known that VBAC is an option provided the details of the previous caesarean are available and there is close monitoring during labour with the ability to proceed to an emergency caesarean if needed [22,23].

Other NCCWCH/RCOG-recommended indicators for caesarean section including multiple pregnancy, mother to child transmission of disease, maternal request, placenta praevia and preterm or small for gestational age [2] were reported as minimal indicators for caesarean section in the South East Asian hospitals audited.

### Use of antibiotic prophylactic

The use of prophylactic antibiotics is recommended to reduce endometritis and wound infection after elective or non-elective caesarean section [24]. This knowledge has been applied in all hospitals of the four South East Asian countries audited, where prophylactic antibiotics were almost always given.

Evidence suggests that prophylactic antibiotics should be administered pre-operatively to result in the lowest risk of surgical wound infection [24]. The variation in timing of antibiotic prophylactic administration between hospitals, and the lack of consistent timing in relation to the type of institution may suggest that some individual hospitals have developed standardized policies for use of antibiotic prophylactics, while some individual health professionals may practice in line with their own preferences at other hospitals.

First generation cephalosporin and ampicillin have been found to be equally effective agents for antibiotic prophylaxis for women who underwent a caesarean [25] and this recommendation was followed in nearly 90% of all cases reviewed in the four South East Asian countries.

Multiple doses of prophylactic antibiotics have been found no more effective than a single dose [25] and are more expensive. It is therefore of interest that our review found that all mothers who underwent caesarean in Indonesia, in one hospital in Malaysia and in one hospital in Thailand, were given multiple doses. Current evidence does not support this expensive practice.

### Advantages and limitations of the study

There is lack of completeness of reporting for deaths and infection within South East Asian hospitals. The reporting may be improved for certain outcomes that may be considered as important quality control or assurance indicators for health care. Many of the SEA-ORCHID data indicators may be recommended for such purpose. The health indicators used were clearly defined and dedicated data staff were trained in their collection. The variation in care practices seen such as for use of resuscitation of the newborn at caesarean section, are likely to reflect different hospital policies as well as differences in casemix.

### Adherence to best practice recommendations

Within nine hospitals in four South East Asian countries, our audit has shown varying and non-structured uptake of evidence-based clinical guidelines and recommendations in relation to caesarean section. This may be due to lack of availability and access to medical journals and reviews and therefore limited dissemination of evidence-based guidelines and recommendations. Availability of access and enablers and barriers to uptake of evidence based guidelines need to be examined at individual institutions. The SEA-ORCHID study plans to conduct a survey of evidence-based practice knowledge and clinical change among maternal and infant health practitioners in South East Asia to explore this issue [16]. It would be of benefit for each institution to develop policies regarding caesarean, and particularly the timing and dosing of administration of prophylactic antibiotics so as to encourage standardized practice and to reinforce that access to knowledge and information is important.

## Conclusion

The baseline rates of caesarean section, associated clinical practices and outcomes varied considerably in nine hospitals of four South East Asian countries comprising Thailand, The Philippines, Malaysia and Indonesia. The most common indications for caesarean delivery were malpresentation, previous caesarean section, cephalopelvic disproportion, and fetal distress. Maternal request remained rare. Giving prophylactic antibiotics was nearly universal, with variations in the timing of administration, (either pre-operatively, after cord clamping, or post-operatively), and variation also in the class and number of doses of antibiotics given. Blood loss during caesarean was commonly estimated to be less then 500 ml. A few women received blood transfusions. Postnatal care for mothers varied widely between hospitals within countries and also across countries.

## Competing interests

The authors declare that they have no competing interests.

## Authors' contributions

MF, DHS and CC contributed to the design of the study. ML and PP cleaned and analysed the data and all authors contributed to the interpretation of data. ME prepared the first draft of the paper. All authors commented on each draft of the paper.

## Pre-publication history

The pre-publication history for this paper can be accessed here:


